# Tree conservation can be constrained by agents from conservation permitting and funding agencies

**DOI:** 10.1080/19420889.2019.1654348

**Published:** 2019-08-19

**Authors:** Thomas E. Marler

**Affiliations:** College of Natural and Applied Sciences, University of Guam, Mangilao, Guam, USA

**Keywords:** Conservation, Cycas micronesica, Guam, military ecology, *Serianthes nelsonii*

## Abstract

Recent conservation actions for *Serianthes nelsonii* Merr. and *Cycas micronesica* K.D. Hill in the Mariana Islands have illuminated some negative consequences associated with ill-informed agents representing permitting and funding agencies. Several cases from the islands of Guam and Tinian are discussed as ineffective conservation examples, and these are countered with two examples of successful conservation approaches. When biologists that act as points of contact for federal permitting and funding agencies do not possess education, knowledge, and experience that is germane to federally listed species, sound science may be marginalized from the conservation agenda. When rapid turnover of federal conservation agents introduces dysfunction, discontinuities in collaborations may thwart success. When lapses in conservation contracts are allowed, short-term extemporary contracting approaches are utilized, and conservation practitioners that lack the ability to include an experimental approach to conservation actions are employed, the co-production of new knowledge to enable decision support tools for future decision-makers may be hindered.

The volume of biology and ecology research devoted to improving decisions during conservation of threatened and endangered tree species in the Mariana Islands has been deficient. Yet copious amounts of federal funds have been and will be spent on terrestrial resource conservation projects during the ongoing military buildup on Guam and Tinian [1]. Co-production of knowledge is crucial in environmental management programs because management decisions continually improve as the new knowledge accumulates [2]. Taxpayers who are paying for the expensive conservation activities in the Mariana Islands deserve to have sound science be the guide for the ongoing conservation agenda, thus the conservation actions would benefit if the co-production of new knowledge could become a formal component of agency decisions.

A diagnosis of the key issues that have limited progress in global plant conservation is warranted [3]. Sharing successful and unsuccessful local case studies with the international community is a vital step toward learning and adapting within conservation and restoration efforts [4]. Discussions of conservation failures are particularly important for helping conservationists avoid counter-productive decisions, and these discussions provide core diagnostic tools for building future conservation program successes [5–7]. For example, weak governance may create institutional confusion or foster corruption that thwarts conservation and restoration successes [8–10]. Additionally, government agents in positions of power may use a top-down assertive approach rather than a collective integrative approach to push an authoritarian agenda that ignores the fundamental principles of research ethics and allows the loss of intellectual property by involved scientists [11–13]. Global conservation efforts are hindered when individuals empowered with making policy decisions are insufficiently equipped to understand the local conservation issues, and similarly hindered when individuals possessing local knowledge and capabilities are marginalized from the conservation process [7].

*Serianthes nelsonii* is an arborescent species from the Mariana Islands with a restricted endemic range of Guam and neighboring Rota [14,15]. The species has been listed as Endangered under the United States Endangered Species Act (ESA) since 1987 [16], and a formal recovery plan has been in place since 1994 [17]. A recent publication illuminated the negative consequences to conservation of this species that resulted from the historical use of extemporary short-term contract approaches rather than an integrated, sustainable conservation approach [18]. A second recent publication discussed how uninformed agents from permitting and funding agencies were critical of the use of pruning as a horticultural tool for *Serianthes* plant production [19]. Yet the collective evidence to date has revealed that pruning of *Serianthes* plants exerts no detrimental influence on plant development if the practitioner is a plant physiologist with an understanding of plant water relations [20], and repetitive pruning of plants in a *Serianthes* nursery greatly improves post-transplant growth and survival [19]. These characteristics of the 25-year Guam case study which dates back to publishing of the *S. nelsonii* recovery plan [17] illuminate a trait that reduces progress on any formal conservation effort: uninformed agents from federal permitting and funding agencies who are entrusted by the public to plan and execute the conservation agenda. Several cases from the islands of Guam and Tinian are discussed as ineffective conservation processes that hinder the co-production of new knowledge, thereby diminishing the decision support toolkit for future decision-makers.

## Permitting agencies, contracting agencies, and conservation practitioners

The biologists within federal permitting agencies such as the United States Fish & Wildlife Service (USFWS) develop conservation protocols, approve practitioners who have applied for permits to work with threatened species, and monitor adherence to the protocols of the ESA guidelines. The biologists within conservation funding agencies such as the United States Department of the Navy (DoN) serve as technical points of contact for practitioners in conservation contracts, enable access to restricted conservation sites, and ensure contract performance objectives are addressed. Contracting officers from funding agencies such as the DoN partner with biologists to define contract objectives, advertise, and select bids from potential contractors.

**Figure 1.**
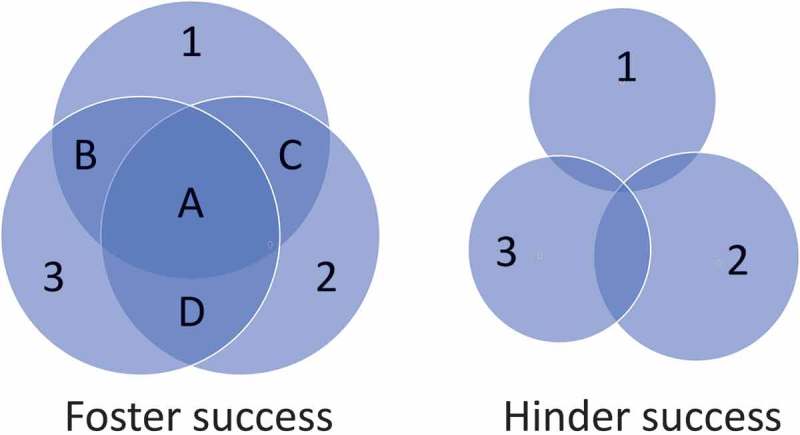
Venn diagram depicting the relationships among conservation permitting agency (1), funding agency (2), and practitioners (3). Size of the sphere for each participant portrays education and knowledge of representatives and adherence to respective responsibilities. A substantial three-way overlap (a) represents relative success of a conservation project (left). To foster success, the permitting agency respects the responsibility to support the practitioner within the guidelines of the permit (b), the two agencies work with synergy to enable the practitioner to perform at a level that matches their capabilities (c), and the funding agency provides resources and removes roadblocks that hinder practitioner performance (d). An example that hinders success may occur when the funding agency demands oversight of the permit compliance, and the permitting agency surrenders that oversight (right). The size of the sphere for permitting agency is greatly reduced due to the decision to abandon the responsibility to support the practitioner. The size of the sphere for the funding agency is reduced due to misfeasance, but the reduction is not as great because the agency has taken over a disproportionately and inappropriately larger role in the conservation process. The size of the sphere for the practitioner is greatly reduced because performance is damaged by the dysfunction between permitting and funding agencies. The overlap of permitting agency and practitioner is minimal, the overlap of permitting agency and funding agency is minimal, and the three-way overlap approaches nil.

The interactions among conservation employees from the permitting agencies, funding agencies, and practitioners define conservation successes or failures. For success, each entity in the triad should employ educated and knowledgeable representatives, should fulfill their entity’s role in the collaboration, and should not commit misfeasance by acting outside the boundaries of their role (Figure 1). Public stakeholders need to believe that conservation projects supported with federal funds are being implemented with the most appropriate approaches based on sound science. The following examples reveal how the trust of taxpayers may be undermined and progress in adaptive management may be damaged when federal conservation employees do not possess the basic knowledge to understand the biology and ecology of the threatened species.

### Case 1. Federal agents devoid of requisite knowledge and experience

#### The facts

*Cycas micronesica* was recently ESA-listed as threatened [21]. The species provides a compelling example of how invasions of non-native species can be devastating in insular settings. The tree was the most abundant species in Guam’s forests in a 2002 survey [22], became host to the armored scale *Aulacaspis yasumatsui* and several other invasive specialist insect species beginning 2003 [23], suffered epidemic plant mortality as a result of the invasions [24], then was listed as Endangered under the International Union for Conservation of Nature (IUCN) by 2006 [25]. USFWS biologists listed this species as a “flowering” plant [26], and the mitigation requirements from the Biological Opinion (BO) for handling *C. micronesica* trees during tree salvage projects associated with military construction activities on Guam included the assessment of survival based on “documented observation that the plant is ready to set fruit or flower” [27].

The USFWS employees who developed these conservation instructions and the DoN biologists who vetted the BO prior to publication had numerous *C. micronesica* publications in the contemporary primary literature available for their review. This accessible literature clearly established the fact that this tree is the only native gymnosperm species in the region. Gymnosperms lack the ability to produce flowers or fruits, facts of which freshmen university biology students are aware. All conservation practitioners who will be contracted by the DoN to fulfill mitigation tasks identified in the BO sections associated with salvage of *C. micronesica* trees will fail because ill-informed federal conservation employees were allowed to impose performance standards that are biologically impossible to meet.

This issue is not restricted to the biologists who directly interact with conservation practitioners, as the administrative and contracting levels of the DoN have also been represented by personnel without germane knowledge and experience in tree biology and ecology. Administrators without appropriate education lack an understanding of the threatened species, so they are not equipped to assess and correct poor decision-making by the biologists. Representatives of the DoN contracting staff have power over conservation contracting decisions, so when these individuals override the biologists’ conservation contract specifications, demand exclusive oversight of contractor choice, and hamper direct communications between contractors and DoN biologists, their actions severely damage conservation success because they are not educated in biology. For example, contracting agents often resort to awarding contracts exclusively on lowest bid and reject the DoN biologists’ advice on awarding contracts based on the best available science.

#### Interpretation

Addressing mitigation of the threats to a federally listed species cannot be achieved in the absence of basic biological knowledge of the species, an issue that is magnified for plants with extremely small contemporary populations [28]. Appropriate expertize is required for conservation success [29]. Therefore, this form of naivety among ill-educated federal conservation employees conveys to the public that the agencies do not understand the terrestrial species that they are obligated to conserve. For example, the primary threat for *C. micronesica* is ubiquitous infestations of non-native insect pests, and this threat is not being addressed with any of the expensive conservation actions being funded during the ongoing military construction activities [30].

### Case 2. Federal conservation employee turnover

#### The facts

The Mariana Islands DoN has exhibited a revolving door history whereby practitioners are forced to report to numerous sequential points of contact during conservation contracts, ensuring failure in continuity for conservation projects. An example is how the DoN has approached recent management of an *ex situ C. micronesica* germplasm collection on the island of Tinian [31]. Following many years of stability with the same technical representative, four different DoN technical representatives were assigned to the project from 2017–2019.

#### Interpretation

When individual participants of conservation projects are changed, the driving motivations and personal agendas will also change, and the collaborations may fail as a direct result of the participant turnover [32,33]. The dysfunction caused by rapid turnover of federal agency personnel damages performance of proficient practitioners and ensures diminished success of terrestrial conservation efforts. The Mariana Islands environmental work has been hampered in myriad ways by the high turnover among professionals with advanced degrees.

### Case 3. Unclear federal agency responsibilities and boundaries

#### The facts

I managed two sequential DoN contracts with the primary objective of increasing our understanding of the ecology of the endangered *S. nelsonii*. The second DoN technical representative assigned to this project used personal interpretations of USFWS permit compliance to reverse work approvals of the first DoN technical representative of the project. The most dispiriting example was a study that directly informed the primary contract objective by collecting necromass in the vicinity of a mature *S. nelsonii* tree. The field work for the study was approved in the proposed Plan of Action for the first contract and the laboratory work for the study was approved in the proposed Plan of Action for the second contract. These two plan approvals were required prior to conducting the actual work. Thereafter, the ongoing work was approved by the DoN in 19 separate performance reports and further approved by the DoN through the payment of 18 separate invoices that detailed the relevant accomplishments. The second technical representative then reversed these 39 distinct DoN approvals of the important conservation work that directly met a contract objective by inaccurately declaring the collection of the necromass was not allowed under the USFWS permit. This contradicts information from the USFWS which indicated collection of dead materials from the vicinity of a federally listed tree was an activity that falls outside the jurisdictional limits of a USFWS permit.

The text of a USFWS permit indicates practitioners are directly responsible to the USFWS for permit compliance, which means a direct relationship that is not filtered through the funding agency. Therefore, mutual respect for the ESA permit would demand resolution of compliance questions through bilateral negotiation between the permitting agency and the approved practitioner. The issues over which the second DoN technical representative diametrically reversed the approvals of the first DoN technical representative were not contract compliance issues, they were permit compliance issues. My request for the USFWS to resolve the permit compliance questions were denied.

Effective communication among all stakeholders must be actively promoted to reach win-win outcomes in conservation [34]. In this case, the DoN committed misfeasance by taking over a conservation contribution that was exclusively the responsibility of the USFWS, and the USFWS committed nonfeasance by abandoning their responsibility to effectively communicate with their approved practitioner about compliance with their permit.

#### Interpretation

The DoN allowed an agent to abuse the rights of a contractor to fulfill a federal contract, thereby failing to adhere to contract specifications. The USFWS committed a violation of their own permit by conspiring with the DoN to impede the taxpayer-funded conservation responsibilities of both agencies. These agent actions modified the graphical conception of conservation success (Figure 1) in a manner that damaged success. Permitting and funding agencies will marginalize competent scientists from their conservation agenda if they elevate the value of inter-agency politics in a manner that inadvertently undermines their obligations to support the practitioners.

### Case 4. Contracting practitioners without required knowledge and experience

#### The facts

The conservation project described in Case 2 is the only local example of conservation efforts that the DoN has showcased in its international profile of success stories[35]. Yet even this highly successful project was damaged by the DoN’s history of hiring contractors that are devoid of the requisite education to understand the biology and ecology of the biological resource being conserved. The first of two times that this has happened was mentioned as an example of unsuccessful conservation actions earlier[1], and occurred because the contracting officer awarded the contract based on minimum bid over the objections of the DoN biological technical representative. Briefly, a large federal contractor with an existing installation maintenance contract was hired to manage this unique cycad germplasm, despite no cycad biology or ecology expertize. The unqualified field workers for this contractor inflicted lethal damage on the plants when soil berms were constructed on each plant to a depth that completely covered the stems. The capable DoN technical representative at that time was willing to become educated on the function of cataphylls to protect the cycad tree’s stem apex from atmospheric conditions, and that these specialized organs were not outfitted with the machinery to remain alive in the wet, pathogen-ridden subterranean ecosystem. This DoN agent used her time and effort to remove the lethal soil berms the practitioner had constructed, an action that saved the abused plants from demise.

#### Interpretation

The lethal horticultural actions were not the fault of the field workers because they were unaware that their actions would kill the plants. The actions were caused by a DoN contracting officer’s decision to hire a lowest bid contractor that did not possess the requisite biology, horticulture, or ecology knowledge of the conserved species. This example reveals how a federal agent’s decisions can use taxpayer funds to damage the agency’s conservation mission.

### Case 5. Multiple approved practitioners accessing the same conservation site

#### The facts

Efforts to fulfill the contracts described in Case 3 were incepted when two ESA permits with multiple approved practitioners were in existence. Actions from some of the other approved practitioners disrupted my laboratory’s *S. nelsonii* ecology contract performance when they disturbed the litter layer underneath the only remaining Guam *S. nelsonii* tree during their search for seeds to expand their nursery stock. Their choice to inflict this disturbance to the conservation site destroyed 28 months of funded work that had been invested to lay the foundation for a litter decomposition study to determine the amplitude of home field advantage for biogeochemical relations of this endangered species.

An ungulate exclusion fence surrounding the tree was installed and all non-native plant species were removed from within the enclosure in 2012. The exclosure fencing was maintained and all non-native plants were removed from October 2012 until February 2015. These efforts re-established the natural biogeochemical cycling of the conservation site in the absence of non-native plant leaf litter inputs and disturbance by non-native ungulates. For the impending litter decomposition study to be valid, the historical unnatural disturbances prior to October 2012 needed to be muted by the lengthy period of passive restoration. The litterbags for this ecology study were ready to be deployed on the site when the other practitioners inflicted the extreme litter disturbance on the site. Initiation of the biogeochemistry study had to be abandoned after more than two years of preparation.

Non-native ungulates damage habitat relations in numerous ways. Guam’s forests have been acutely damaged by non-native pigs (*Sus scrofa* L.) descended from introductions in the 1600s [36], and deer (*Rusa marianna* Desmarest) descended from introductions in the 1700s [37]. Exlosures of these Guam ungulates have revealed restoration can be rapid [38,39], and soil chemical traits are among the important ecological processes that are restored following ungulate exclosure [40,41]. Physical disturbance of the litter and soil is one means by which non-native ungulates damage ecosystem processes such as nutrient cycling [42–46]. Moreover, the Integrated Natural Resources Management Plan (INRMP) that was guiding all DoN activities at this time was published in 2012 [47], and stated that an ecosystem approach would guide management to “restore and maintain ecological processes” that included “nutrient cycles.” The unqualified practitioners who damaged the ecology of the conservation site by disturbing the litter layer failed to comply with the guidelines of the INRMP.

Photographic evidence of the litter disturbance caused by the other practitioners was provided to the appropriate DoN technical representative along with a request to intervene and protect the contract performance from this damage. Following three months with no action from the DoN technical representative, a second formal request for intervention was submitted. These two formal requests garnered no action to protect the work that complied with the objectives of the conservation contract.

#### Interpretation

Although every approved practitioner retains equal rights of access to ESA-listed specimens, practitioners do not have a right to cause ecological damage that prevents another practitioner’s contract performance. Federal agencies cannot expect conservation successes if they allow their conservation employees to ignore requests to intervene when unqualified practitioners directly damage the ecology of an ESA-listed species. To complicate the situation more, the permittee for the unqualified practitioners that damaged the site was the USFWS, so the permit issuer was also the permittee. These sorts of obfuscated responsibilities and blended agency roles cause conflicts of interest, which lead to confusion that diminishes conservation success.

### Case 6. Lapses in conservation funding

#### The facts

The focus on short-term extemporary contracting approaches and a failure to appreciate the direct damage to living organisms caused by lapses in monitoring and maintenance has been a consistent component of conservation efforts by the DoN. By 2017 when I was contracted to develop a formal plan for long-term management of the Tinian *C. micronesica* germplasm, two consequential lapses in funding had occurred since the project was initiated in 2006. Both of these gaps in maintenance caused an increase in mortality of the expensive *ex situ* germplasm, a fact that was reported to the DoN. The formal plan called for monthly maintenance activities to sustain viability of this *ex situ* collection. Subsequently, a third period with no funded maintenance activities was perpetrated in 2018 and 2019 and my team’s request to monitor the germplasm without being paid was denied by the DoN.

#### Interpretation

Failures to provide consistent maintenance and monitoring of living resources impede agency obligations to conserve the resource. The decision to establish a permanent *ex situ* germplasm collection of an ESA-listed tree species is difficult to justify if that decision fails to include an enduring mechanism to sustain the minimum amount of effort required to refrain from killing the germplasm. The window of time in 2018 and 2019 with no funded management was greater in duration than the first two episodes with no maintenance. Therefore, the plant mortality in 2019 and 2020 will greatly exceed that following the first two examples of agency-induced mortality. Taxpayers deserve for agencies to administer federal conservation funds more wisely.

### Case 7. Formal data management system required

#### The facts

The DoN germplasm in Tinian is one of three *ex situ* collections that were established in 2006 with the goal of conserving Guam’s *C. micronesica* genetic diversity[35]. The germplasm located in the Thailand and Florida sites has been maintained without interruption by international experts on cycad biology, and the records for each individual plant within these *ex situ* collections have been stored in BG-Base, a platform developed in conjunction with the Center for Plant Conservation, and BRAHMS, a platform developed at the University of Oxford. To date, the DoN has not funded a formal data management system for the Tinian *C. micronesica* data or information from the other recent large-scale conservation projects associated with the military buildup.

#### Interpretations

Data storage in some form of information management platform is required for conservation efforts to be of value [3]. The Thailand and Florida *C. micronesica ex situ* collections that are being professionally curated at botanical gardens will remain of crucial importance for reintroductions to Guam in the future. After the current curators who manage the accumulating data retire, these botanical gardens will recruit new curators with appropriate cycad expertize to continue the data management. This sustainable approach to safeguarding the future of a botanical garden’s germplasm [48,49] assures conservationists that the Thailand and Florida collections are effectively safekeeping Guam’s genes. In contrast, the DoN’s decisions to hire practitioners without cycad expertize, the repetitive lapses in funded management activities, and the lack of concern to fund a formal data management system minimizes the future value of the Tinian germplasm.

## Success is available if empowered individuals desire it

A long-term, consistent, success-oriented approach to decision-making in conservation and restoration projects is achievable, but only if the empowered decision-makers desire success. Two examples serve as success stories for federal permitting and funding agencies.

Example 1. The United States Forest Service (FS) has funded numerous conservation projects associated with the threats to *C. micronesica*. The FS employs technical representatives with appropriate education such that they understand the underlying issues and can digest the performance information from contracted practitioners. The FS maintains these resource persons for many sequential contracts, ensuring successful continuity of expertize among the collaborative conservation projects. The FS biologists are embraced by FS contracting officers as the agents with pertinent education and they work closely together throughout the entire contractor selection process. This approach by the contracting officers ensures the appropriate level of biological education guides all contracting decisions.

I managed seven contracts on *C. micronesica* conservation for the FS from 2005–2019, and the same expert who possesses a Bachelor of Science degree in biology and Master of Science degree in entomology has served as my technical representative. Consistent monitoring is often given low priority in conservation[3], but the FS contracts have ensured uninterrupted monitoring of the *C. micronesica* and *A. yasumatsui* populations throughout the native range of the plant. At times the FS technical representative initiated new contracts before the termination of existing contracts as a means of ensuring no lapses in monitoring would occur. Indeed, ecosystem changes can be misunderstood in insular settings if consistent monitoring is not used[50]. This wise approach of a funding agency enabled the accumulation of the only existing long-term *C. micronesica* survival data set throughout three islands and has resulted in numerous peer-reviewed articles that are available to inform future *C. micronesica* conservation decisions [51–68].

The FS has openly embraced the need for various stakeholders within the community to participate in management decision-making. The manner in which these extra-government participants contribute to FS decisions and how those contributions modify FS structures and processes have been studied with an empirical approach [69].

Example 2. A highly successful approach to conservation planning can be found in well-conceived conservation efforts of the Seychelles [70]. In this case, the government conservation employees who were empowered to plan and implement the conservation agenda initiated the conservation efforts of the coco de mer palm (*Lodoicea maldivica* Gmelin) by including accomplished scientists as a beginning step, enabling the scientists to implement adaptive management research, allowing them to publish the results in the primary literature, then culminating the process by crafting conservation decisions based on the scientific publications [71]. The approach has resulted in the generation of informative multi-year data sets that have greatly improved conservation decisions [72,73].

## Final thoughts

The 25-year-old *S. nelsonii* recovery plan [17] called for research to inform conservation management decisions. Then practitioners who demonstrated no evidence of an ability to conduct this mandated research were permitted and funded, such that no peer-reviewed publications based on an experimental approach to conservation actions were generated during the first two decades of the recovery plan. These selections of practitioners were permitting and funding agency decisions that guaranteed the research component of the recovery plan would not progress. The recovery plan also called for establishment of *in situ* plantings containing thousands of mature *S. nelsonii* trees showing sustainable regeneration and recruitment without active management. To date, not a single tree has been nurtured to maturity and little progress has been made toward reaching these goals. *In situ* regeneration and horticultural propagation are not limitations to species recovery [18–20]. In contrast, *in situ* recruitment to the sapling stage and successful establishment of nursery stock after out-planting from a nursery are acute limitations. The worldwide population of ~121 trees at the time of the recovery plan [74] has declined to ~33 trees [20], so almost three-fourths of the taxon’s genetic diversity has already been lost. To my knowledge, no experimental work conducted by accomplished scientists is currently funded to uncover the causes of these recruitment limitations, so the unexceptional progress of the past 25 years concerning *in situ* population expansion will likely remain mediocre into the future.

The ongoing conservation actions with *C. micronesica* are also unimpressive. This species devolved from the most populous tree on Guam in 2002 [22] to being Red-listed as endangered by the IUCN in 2006 [25]. Recent and ongoing conservation decisions by permitting and funding agencies are focused on land-use change, which is a nominal threat that is not among any of the primary threats to this species [30]. To my knowledge, no conservation work is being funded to mitigate the primary threats to the unique tree species.

A decision by the USFWS and DoN to adopt the conservation approaches modeled by the FS and the Seychelles conservation agencies would likely reverse the established culture of mediocre tree conservation on Guam. Following are suggestions that may aid in that effort.

### Federal conservation employees

Federal permitting agencies such as the USFWS are entrusted by the taxpayers to use state-of-the-art approaches to fulfill the agency’s conservation mission. This trust is undermined when the agencies empower ill-informed biologists with the responsibility to dictate how conservation actions can and cannot be carried out. Successful conservation outcomes cannot be expected of competent practitioners if the USFWS reveals in federal guidelines that the biology of the threatened species is not understood by the agency.

Federal funding agencies involved in natural resources conservation are expected by taxpayers to hire conservation employees with a germane university degree and requisite knowledge and experience. Performance of contracted practitioners cannot be effectively evaluated when the federal points of contact do not understand the basic biology and ecology of the threatened species. Conservation success cannot be expected if contracting officers with no relevant biology education reject the inputs of agency biologists and decide how contracts are awarded without regard to best available science.

Conservation agencies should determine if their guidelines and actions are appropriate to achieve conservation success [3]. Scientists are fundamental to solving environmental challenges, so conservation agencies should consider all approaches to enable effective research and information dissemination [75]. Alternatively, the contributions by scientists may be diluted by government agency decisions that create a culture where ecologists feel unsafe to convey their intellectual property to decision-makers [76]. As a means of addressing this, Roux et al [77]. argue that conservation agencies should bring scientists into their staffing pattern as a means of increasing the respect for co-production of knowledge during conservation projects. For funding agencies where this wise approach to conservation is not endorsed by the decision-makers, the minimum standards should include conforming to what successful federal funding agencies do by hiring federal agents with appropriate education and proven knowledge that directly relates to the terrestrial resource that is being conserved [29].

### Practitioner qualifications

The responsibility to conserve living organisms must be grounded in an understanding of those organisms. This holds true for the practitioners more so than any of the other participants in formal conservation programs, as these are the individuals who can use naivety to damage or kill the living resource. Conservation projects in the Mariana Islands have been managed without regard for these realities, creating a culture where the co-production of new knowledge is not a recognizable component of the conservation agenda. This history does not conform to recommendations for managing species at risk within military installations, which calls for scientifically-based management that focuses on the greatest threats [78]. Employing conservation practitioners with the proven skills of contributing to the primary literature as an initial step of adaptive management, in accordance with the Seychelles example [71], would point the trajectory of Mariana Islands tree conservation toward conformity with the recommendations of the Center for Plant Conservation [49].

### Institutional record-keeping

Conservation projects with living organisms cannot successfully inform future conservation decisions without a long-term commitment to sustained monitoring of the status of the organisms and maintaining the accompanying records in a legitimate platform. To date, the DoN has never funded a record-keeping system for the valuable *C. micronesica ex situ* germplasm on Tinian Island. Establishing a bona fide record-keeping system for the duration of the military buildup’s terrestrial damage is a reasonable expectation by taxpayers, as botanical gardens throughout the world already do this as a foundation to their conservation efforts [48,49].

### Co-production of new knowledge

International recommendations for plant conservation include the use of non-destructive experimental approaches within conservation projects [49]. These recommendations are exemplified by international trends in environmental management that include co-production of new knowledge during management activities as a means of legitimizing conservation actions [2,79]. These international recommendations have been conspicuously lacking from conservation projects funded by the DoN in Guam and Tinian. The case of *S. nelsonii* is an example. This tree was the only ESA-listed endangered plant species from the Mariana Islands during the first four decades of the ESA. Despite a call for more research in the 1994 recovery plan [17], numerous federal contracts, and many approved practitioners, no experiment-driven articles were published for the first two decades of the recovery plan. Access to historical ecological evidence is crucial for guiding future restoration work of all kinds, especially when a social science component is involved [80]. Ecological projects such as the tree conservation projects in the Mariana Islands rely on synergistic interactions among permitting agents, funding agents, and practitioners, and are quintessential examples of long term social-ecological programs. Valuing co-production of new knowledge and enabling capable practitioners who possess the intellectual property rights of that new knowledge to publish in peer-reviewed journals should be promoted to safeguard future access to information rather than marginalized from the conservation agenda.

### The military buildup

Environmental and conservation efforts are often carried out within the context of a military setting, creating a restrictive culture for involved scientists [81]. The environmental destruction that is accompanying the colossal contemporary military buildup is an example of how Guam’s permanent civilian population has been marginalized from consequential environmental decisions, as the original agreement between the U.S. and Japan was formalized without the involvement of a Guam representative [1,82]. Against this historical backdrop, the consensus-building approach of pursuing respectful input of all stakeholders has not found its way into federal conservation decisions involving Guam’s terrestrial resources, possibly because this approach does not conform to the top-down peculiarities that define a military culture [83]. Indeed, the “command-and-control” leadership philosophy that is prevalent within a military culture is counter-productive within conservation programs and leads to failed conservation outcomes when it is adopted by decision-makers [29]. Alternatively, empowered conservation decision-makers generate success by embracing a supportive framework that focuses on cooperative teamwork and communications [84]. Improving equity among all participants is viewed as an enabler of successful outcomes for conservation decision-makers [85].

Billions of dollars will be spent during the military buildup on Guam and Tinian in the near future. The environmental destruction has already been and will continue to be substantial. The established DoN culture of employing ill-educated technical representatives creates a working framework where the most important federal liaison does not possess the requisite biology or ecology knowledge. Allowing frequent turnover of conservation employees adds gratuitous dysfunction and ensures a maximum of discontinuity among conservation projects. Hiring practitioners with no proven ability for co-production of new knowledge guarantees that the best available science mandate of the ESA [86] will not become a perceptible component of the conservation efforts during the expensive military buildup. Failing to plan and forecast appropriately in a manner that causes lapses in conservation contracts will continue to undermine the DoN’s conservation obligations. These federal agency approaches to managing conservation of the terrestrial resources of the Mariana Islands reject contemporary international recommendations for conservation and restoration programs.
